# Magnitude of episiotomy practice and associated factors among women who gave birth at Hiwot Fana Specialized University Hospital, Eastern Ethiopia

**DOI:** 10.3389/fgwh.2022.911449

**Published:** 2022-10-10

**Authors:** Habtamu Bekele, Dawit Tamiru, Adera Debella, Alemeshet Getachew, Ephrem Yohannes, Magarsa Lami, Abraham Negash, Henock Asfaw, Indeshaw Ketema, Addis Eyeberu, Sisay Habte, Bajrond Eshetu, Tamirat Getachew, Sinetibeb Mesfin, Bekelu Birhanu, Helina Heluf, Haregeweyn Kibret, Belay Negash, Adisu Alemu, Yadeta Dessie, Bikila Balis

**Affiliations:** ^1^School of Nursing and Midwifery, College of Health and Medical Sciences, Haramaya University, Harar, Ethiopia; ^2^School of Public Health, College of Health and Medical Sciences, Haramaya University, Harar, Ethiopia; ^3^School of Nursing and Midwifery, College of Health and Medical Sciences, Ambo University, Ambo, Ethiopia

**Keywords:** episiotomy, magnitude, parity, induced labor, medical conditions, Ethiopia

## Abstract

**Background:**

Episiotomy is an intentional surgical incision made on the perineum with the aim of enlarging the introits during the second stage of labor or just before delivery of the baby. It sometimes also interferes with the mother's comfort during the postpartum period and has associated complications especially when it is done without indication. However, there is limited information regarding episiotomy practice in the study area.

**Objective:**

This study aimed to determine the magnitude of episiotomy practice and associated factors among women who gave birth at the Hiwot Fana Specialized University Hospital, Eastern Ethiopia, 2021.

**Methods:**

A facility-based cross-sectional study was conducted among 408 systematically selected mothers who gave birth at the Hiwot Fana Specialized University Hospital, from 1 January to 30 December 2021. Datas were collected from delivery medical records using a pretested checklist. The extracted data were checked, coded, and entered into the Epi-data version 3.1 and exported to the STATA version 16 software for analysis. Binary logistic regression was fitted to identify factors associated with episiotomy practice. *P*-values < 0.05 were considered to declare the presence of statistical significance.

**Results:**

The overall prevalence of episiotomy practice was found to be 43.4 % (95% CI: 38.7, 48.9), and mediolateral was the most commonly practiced episiotomy type (41.4%). Parity [AOR: 6.2; 95% CI (3.8–17.6)], 1st min Apgar score [AOR: 1.6; 95% CI (1.04–2.67)], presence of maternal medical disease [AOR: 3.3; 95% CI (1.09–6.9)], and induced labor [AOR: 1.6; 95%CI (1.12, 4.13)] were significantly associated with the episiotomy practice.

**Conclusion:**

The prevalence of episiotomy practice in the study area was high. Parity, presence of maternal medical disease, induction of labor, and 1st min APGAR score were significant factors associated with episiotomy practice. Considering the presence of appropriate indications or preventing unjustifiable indications, can help to reduce the current high practice rates.

## Introduction

An episiotomy is one of the widely practiced obstetric interventions performed to minimize the risk of severe tears or to enlarge the birth outlet during childbirth at a time when the fetus's head descends (1, 2). It helps in avoiding potential intracranial damage to the infant, prevents low Apgar score, and avoids severe and irregular perineal tears. The procedure also helps to prevent the relaxation of pelvic floor muscles and the incidence of cystocele and rectocele (3).

Even though seven different types of episiotomy have been reported in works of literature, two major types are routinely used: median and mediolateral episiotomy (2, 4, 5). Mediolateral episiotomies are most commonly performed in Europe, whereas midline episiotomy is common in the USA (6). World Health Organization (WHO) expressed a lack of evidence to perform routine or liberal use of episiotomy for women undergoing spontaneous vaginal birth and recommended restrictive episiotomy, despite its particular rate not being suggested (7).

The practice of episiotomy is highest in Latin America and lowest in Europe. The prevalence ranges from 9.7% in Sweden to 100% in Taiwan. For nulliparous women, a range from 63.3% in South Africa to 100% in Guatemala has been recorded, indicating an overall greater likelihood for primiparous to receive an episiotomy at birth (8). In Africa, the rates of episiotomy also varied: Rwanda reported 80.1% of episiotomy practice among primiparous women (9), and Ethiopia listed among the highest (10–14). In Ethiopia, findings from studies conducted in different parts of the country revealed that the magnitude of episiotomy is over 30%, and the practice was reported to increase up to 2.3-folds more in rural parts of Ethiopia (10, 14).

Most of the consequences of episiotomy affect the laboring mothers, greatly impacting their quality of life and leaving them with an unpleasant childbirth memory. A report showed that episiotomy alone had caused around 9 and 8% of primary postpartum hemorrhage and maternal sepsis, respectively (15). Findings also indicated that the procedure has increased the risk of subsequent pelvic floor dysfunction, fecal and urinary incontinence, spontaneous perineal tear, sexual dysfunction, and fetal head injury (16–18). Episiotomy is also associated with a higher incidence of perineal pain in the immediate postpartum period, which predisposes them to a risk of psychological morbidity and stress urinary incontinence at 6 weeks postpartum (19, 20). Studies also indicate routine episiotomy practice has undue adverse effects, such as extreme perineal trauma, or prolapse of the pelvic organ compared to restrictive episiotomy usage (21–23).

In addition, episiotomy is done to prevent severe perineal tears, even though it has been related to an increased risk of severe bleeding, wound hematoma, dyspareunia, increased discomfort, anal postpartum incontinence, and infection, implying minimal practice (23, 24). Therefore, indications for routine episiotomy are not well-supported or restrictive use of episiotomy is advocated (25, 26).

Reducing the non-evidence-based practice of episiotomy is a global target in improving maternal health and their birthing experiences (27, 28). A number of strategies have been suggested by international leading associations and communities. Continuous quality improvement programs, obstetric interventions, including choosing different positions of birth, and setting an acceptable rate of episiotomy (10%) for normal deliveries were some of them (29–31).

Factors like obstetric procedure, maternal and fetal conditions, increase in age of women, prim-parity, malposition of fetus, instrumental delivery, and vaginal breech delivery have shown an association with why usually episiotomy is practiced (32–34). Despite plenty of research evidencing against liberal use of episiotomy and its related complications, the rate continues to rise in many developing nations. Epidemiologically reliable prevalence and associated factors of episiotomy practice are not well-studied in the study area. Therefore, this study aimed to determine the magnitude of episiotomy and factors associated with women who gave birth in HFSUH, Eastern Ethiopia, which might help reduce the non-evidence-based practice of episiotomy and associated complications in lower-income settings.

## Method and materials

### Study design, setting, and period

A facility-based quantitative cross-sectional study was conducted at HFSUH, a tertiary referral center affiliated with the College of Health and Medical Sciences, Haramaya University, Ethiopia. The hospital provides both general and specialist services for about 3.8 million populations in Eastern Ethiopia with more than 4,000 deliveries per annum. During the study period, the unit was run by seven consultants, 29 residents, and 43 midwives. A neonatal intensive care unit (NICU) equipped with essential equipment for caring neonates with special care needs is available. The study recruits women who gave birth from 1 January 1 to 30 December 2021.

### Population

Women who gave birth through vaginal delivery at the Hiwot Fana Specialized University Hospital were the source population. Women who gave birth through vaginal delivery at the Hiwot Fana Specialized University hospital from 1 January to 30 December 2021 were the study population. The study excluded records with incomplete information.

### Sample size determination

The minimum sample size required for this study was determined using a single population proportion formula with assumptions of confidence level at 95% = 1.96, a margin of error (*d*) = 0.05, and a reasonable proportion of episiotomy practice (41.4%) from the previous study in conducted Tigray (12).


$$\begin{array}{l} n=\frac{{\left(Z\alpha /2\right)}^{2}p\left(1-p\right)}{d^{2}}=\frac{{\left(1.96\right)}^{2}\left(0.414\right)\left(1-0.414\right)}{{\left(0.05\right)}^{2}}=371 \end{array}$$

Then, after adding contingency for incompleteness (10%), the total sample size calculated for this study was 408.

### Sampling technique

The unique medical record number of women who gave birth by vaginal delivery obtained from the registration book was used to create the sampling frame. The total number of women who gave birth by vaginal delivery (2,548) during the study period was divided by the total sample size (408) to obtain the fixed periodic interval value (K-value) of approximately 6 (6th). A systematic random sampling technique was utilized to select a medical record by the determined K-value.

### Data collection

Data were collected using the data extraction checklist developed after reviewing relevant literature (10, 14, 35). The extraction sheet addressed the socio-demographics, pregnancy, labor and delivery, and postnatal characteristics. Data were collected by six BSc holder midwives and supervised by two public health specialists (MPH).

### Variables

The outcome variable of the study was episiotomy practice. The independent variables included socio-demographic factors, pregnancy, labor, delivery, and postnatal characteristics.

### Data quality control

To assure the quality of data, training was given to data collectors and supervisors on the objective of the study for 2 days. A pre-test was done on 5% of the sample size before the actual data collection. The checklists were modified based on the pretest result, repetitive ideas, and ambiguous questions were corrected, and the modified checklists were used for the final data collection. Data were checked for completeness, accuracy, clarity, and consistency by the principal investigator and supervisors before data entry into the software. Double data entry was done for its validity and compared to the original data. Simple frequencies and cross-tabulation were done to check for missing values and variables.

### Data processing and analysis

The collected datas were checked for completeness, cleaned, edited, coded, and entered into the Epi data version 3.1 to minimize logical errors and design skipping patterns. Then, the datas were exported to STATA version 16 for analysis. The assumptions for binary logistic regression were checked. Bivariate and multivariable logistic regression analyses were done to check the association between the practice of episiotomy and each independent variable. All variables with *P*-value < 0.2 in the bivariate analysis were included in the final model of multivariate analysis to control all possible confounders and the variables were selected by step-wise selection. The goodness of fit was tested by the Hosmer-Lemeshow test (*P*-value = 0.561). The multi-co-linearity test was carried out to see the correlation between independent variables by using standard error and variance inflation factor (VIF). Adjusted odds ratio along with 95% CI was estimated to identify factors associated with episiotomy practice. A *P*-value < 0.05 was considered to declare the presence of a statistically significant association.

### Ethics approval and consent to participate

Ethical approval was obtained from Haramaya University, College of Health and Medical Sciences, Institutional Health Research Ethics Review Committee (IHRERC). Data collection began after obtaining informed, voluntary written consent from the head of the institution. Throughout the study period, the confidentiality of the data was strictly maintained through a collection of anonymized record only.

## Results

### Socio-demographic characteristics

In this study, 408 records of mothers who gave birth have been reviewed. Among the majority of the study participants, 216 (52.9%) were between 15 and 24 years old and the mean age was 25.1(+/−4.3 SD). Most of them were urban residents (57.1%). About 235 (57.6%) of the study participants were Muslims by their religion. Only 26 (6.4%) mothers had medical conditions, including hypertensive, metabolic, cardiac, and respiratory disorders (Table 1).

**Table 1:** Socio-demographic characteristics of women who gave birth in the Hiwot Fana Specialized University Hospital, Eastern Ethiopia, 2021.

**Variable**	**Frequency (*n* = 408)**	**Percentage (%)**
**Age in years**		
15–25	216	52.9
26–34	177	43.4
>35	15	3.7
**Residence**		
Urban	233	57.1
Rural	175	42.9
**Marital status**		
Married	399	97.7
Not Married	9	2.3
**Religion**		
Muslim	235	57.6
Protestant	27	6.6
Orthodox	146	35.8
**Presence of maternal disease during pregnancy**		
Yes	26	6.4
No	382	93.6

### Pregnancy, labor, and delivery related characteristics

From the total reviewed mothers' records, 274 (67.2%) had antenatal care (ANC) follow-up on index pregnancy. Regarding birth experience, 76 (18.6%) mothers were pregnant for the first time. In addition, 292 (71.6) had given birth through SVD previously. Among mothers who had previous vaginal births, 99 (24.2%) had previous episiotomy experiences. About 328 (80.4%) had spontaneous labor onset (Table 2).

**Table 2:** Pregnancy, labor, and delivery characteristics of women who gave birth in the Hiwot Fana Specialized University Hospital, Eastern Ethiopia, 2021.

**Variable**	**Categories**	**Frequency**	**Percentage %**
Parity	Primi gravida	76	18.6
	Multi gravida	332	81.4
Previous mode of delivery	C/S	35	8.6
	SVD	292	71.6
	Instrumental	4	1.0
FANC attending	Yes	274	67.2
	No	134	32.8
GA at delivery	<37 wk	41	10
	37–42 wk	339	83.1
	>42 wk	28	6.9
Onset of labor	Spontaneous	328	80.4
	Induced	80	19.6
Utilization of oxytocin during Labor	Yes	54	13.2
	No	354	86.8
Fetal presenting part during labor	Vertex	339	83.1
	Breach	25	6.1
	Other	44	10.8
Duration of 2nd stage of labor	<30 min	138	33.8
	30 min-1 h	211	51.7
	>1 h	59	14.5
Mode of delivery	SVD	325	79.7
	Vacuum delivery	56	13.7
	Forceps	27	6.6
Presence of meconium	Yes	36	8.8
	No	372	91.2
Presence of Fetal distress	Yes	5	1.2
	No	403	98.8
Number of pregnancy	Singleton	399	97.7
	Multiple	9	2.3

### Birth outcome and postnatal characteristics

This study showed that 142 (34.8%) have the 1st min Apgar score of <6. The presence of meconium was identified in 36 (8.8%) newborns. Furthermore, around 88.2% have birth weights of 2,500−3,999 g.

### Episiotomy practice

The overall prevalence of episiotomy practice was 43.4% (95%CI; 38.7, 48.9). About 101 (24.7%) indicated secondary to tight perineum, while 31 (7.5%) indicated a prolonged second stage of labor. The most commonly practiced type of episiotomy was mediolateral that accounted for around 41.4% (169) and 95.4% of all episiotomies done.

### Factors associated with episiotomy practice

On bi-variate analysis, variables like age, the onset of labor, presence of maternal medical disease, duration of the second stage of labor, use of oxytocin during labor, Hx of the previous episiotomy, gravidity, and the Apgar score at the 1st min had *p*-value score of <0.2 and entered into the multivariable regression analysis. In the multivariable logistic regression analysis, parity, 1st min APGAR score, the presence of maternal medical disease, and induction of labor were significantly associated with episiotomy practice at a *p*-value of < 0.05 (Table 3).

**Table 3:** Factors associated with episiotomy practice among mothers who gave birth at the HFSUH, 2021.

**Variable**	**Category**	**Episiotomy practice**		**COR 95% CI**	**AOR 95% CI**	***P*-value**
		**Yes%**	**No%**			
Parity	Primi para	65 (85.5)	11 (14.5)	11.6 (5.9, 19.1)	**6.2 (3.8, 17.6)**	**0.001**
	Multi Para	112 (33.7)	220 (66.3)	1		
Presence of maternal medical disease	Yes	21 (80.8)	5 (19.2)	6 (2.2, 10.4)	**3.3 (1.09, 6.9)**	**0.001**
	No	156 (40.8)	226 (59.2)	1		
Previous Episiotomy	Yes	33 (33.3)	66 (66.6)	1	1	
	No	144 (46.6)	165 (53.4)	1.17 (1.08, 2.8)	1.1 (0.61, 1.9)	0.634
Onset of labor	Spontaneous	129 (39.3)	199 (60.7)	1		
	Induced	48 (60)	32 (40)	2.3 (1.41, 3.81)	**1.6 (1.12, 4.13)**	**0.041**
Use of oxytocin during labor	Yes	34 (63)	20 (37)	1		
	No	143 (40.4)	211 (59.6)	2.5 (1.3, 4.5)	0.45 (0.15, 1.3)	0.141
Duration of 2^*nd*^ stage of labor	30 min	42 (30.4)	96 (69.6)	1	1	
	30 min-1 h	94 (44.5)	117 (55.5)	1.8 (1.1, 2.8)	1.2 (0.8, 2.1)	0.292
	>1 h	41 (69.5)	18 (30.5)	5.2 (12.6, 10.1)	1.1 (0.46, 2.7)	0.793
1^*st*^ min APGAR score	<6	74 (52.1)	68 (47.9)	1.7 (1.1, 2.5)	**1.6 (1.04, 2.67)**	**0.030**
	>/=7	103 (38.7)	163 (61.5)	1	1	

## Discussion

More than two-fifth of women incurred episiotomy during delivery, and mediolateral was the most commonly (41.4%) practiced episiotomy type. In addition, being primigravida, 1st min Apgar score <6, presence of maternal medical disease, and induction of labor increased the odds of episiotomy practice.

The prevalence of 43.4% (95%CI; 38.7, 48.9) episiotomy practice in the current study is consistent with other findings found in Northwest Ethiopia 44.5% (11), Shire, Ethiopia 41.4% (12), Addis Ababa, Ethiopia, 40% (35), Kano, Nigeria 41.4% (36), Iran 41.5% (37), and studies conducted in developing countries, 36–40% (38).

On the other hand, the current finding is found to be lower than studies conducted in Addis Ababa, Ethiopia (65%) (39), Arbaminch, Ethiopia (68%) (14), Uganda (73%) (40), and in northern Nigeria (89.3%) (41). Similarly, several studies done in different hospitals expose a higher prevalence of episiotomy between 69.2 and 96.2% among primigravid women (18, 42, 43). However, the current result of the prevalence of episiotomy was found higher than study conducted in Jimma 25% (44), Vietnamese-born women (29.9%) (45), Nigeria (21%) (16), Brazil 29.1% (46), Nepal 22% (47), East African women 30% (48), and Mizan Aman 30.6% (33). This difference might happen due to the dissimilarity in settings of the study participants, study period, different sample sizes, sampling techniques, and characteristics of the study population. The elevated prevalence in the current study might be due to the characteristics of the study participant at a specialized University Hospital. The majority of the pregnant mothers who gave birth here were from the category of high risk, complicated cases, and had to go through various obstetric interventions, which might increase the jeopardy of episiotomy.

In congruent with facility-based studies conducted in Addis Ababa, Ethiopia (35, 39), Brazil (49), Nigeria (50), and Uganda (40), primigravid women were more likely to have episiotomy than multiparous women. The study conducted in Uganda indicated that a primigravid mother was four times more likely to have an episiotomy than a multiparas mother (40). This may be due to the perineum muscle of the primigravid mothers being tighter than multiparas women, and due to this, the birth attendant tends to perform a liberal episiotomy.

Mothers of babies with a 1st min Apgar score of <6 were 1.6 times more likely to experienced episiotomy than babies with ≥7 Apgar score. A similar finding was reported from the study conducted at Petrolane, Brazil, which found newborns with lower Apgar scores were two times more likely to happen in the episiotomy group (51). Another study in Ethiopia found that episiotomy happened 5 times more likely among mothers who had low Apgar score bay than those with normal Apgar scores (12). A study conducted in Iran showed a significant mean difference in the 1st-min Apgar score (8.21 ± 0.77) in the episiotomy group and (8.86 ± 1.09) in the non-episiotomy group (37). Additionally, another study result indicated that 21.7% of infants in the episiotomy group and 15.1% in the non-episiotomy group had Apgar scores <7, with a significant difference between them (52).

This study indicated that mothers who had medical diseases were 3.3 times more likely to experience episiotomy than mothers who have no medical disease. This finding is similar to the study done so far in Ethiopia (34). However, a study conducted in Brazil revealed that maternal disease specifically hypertensive syndrome showed a significant association with episiotomy performance (49). This is probably because the birth attendant might think that performing an episiotomy will decrease the duration of labor and delivery particularly in mothers with medical diseases, who are at higher risk for post-partum complications (53).

Furthermore, our study revealed that mothers who gave birth by induced labor had higher odds of episiotomy exposure than those who gave birth by spontaneous onset. This is similar to findings from other studies conducted in Latin America (1), Brazil (18), Iran (37), Northwest Ethiopia (11), and Shire town Ethiopia (34). The possible ground may be if labor does not begin and advance spontaneously, it may not bring perineal muscle's physiological lessening and this could call for perineal intervention (54). Induced labor can also be high in strength and frequency of uterine contractions that may result in non-reassurance fetal heart rate patterns that may need an episiotomy to be applied by the birth attendant in an attempt to decrease the length of labor (55).

### Limitations of the study

This study has some limitations to consider while interpreting the findings. The cross-sectional nature of the study design prevents temporal relationship inference. In addition, the use of records hinders complete information.

## Conclusion

The practice of episiotomy is high in the study area. More than two-fifth of women who gave birth incurred episiotomy during delivery. Parity, 1st min Apgar score, presence of maternal medical disease, and the induced onset of labor were significantly associated with the practice of episiotomy. It is imperative to reduce the rate of episiotomy to improve the well-being and quality of life of women. Considering the presence of appropriate indication or preventing unjustifiable case selection, especially on nulliparous mothers, and induction of labor may help reduce the current high practice rates. In addition, designing programs that improve maternal health conditions to prevent medical diseases, which may complicate pregnancy, would be beneficial.

## Data availability statement

The raw data supporting the conclusions of this article will be made available by the authors, without undue reservation.

## Ethics statement

The studies involving human participants were reviewed and approved by Haramaya University, College of Health and Medical Sciences, Institutional Health Research Ethics Review Committee (IHRERC). The head of institution provided written informed consent to allow data collection for this study.

## Author contributions

All authors listed have made a substantial, direct, and intellectual contribution to the work and approved it for publication.

## Funding

This study was funded by Haramaya University. The funding body has no role in the design of the study and collection, analysis and interpretation of data, and in writing the manuscript.

## Conflict of interest

The authors declare that the research was conducted in the absence of any commercial or financial relationships that could be construed as a potential conflict of interest.

## Publisher's note

All claims expressed in this article are solely those of the authors and do not necessarily represent those of their affiliated organizations, or those of the publisher, the editors and the reviewers. Any product that may be evaluated in this article, or claim that may be made by its manufacturer, is not guaranteed or endorsed by the publisher.

## Abbreviations

ANC: Antenatal care
AOR: Adjusted Odds Ratio
C/S: Cesarean Section
COR: Crude Odds Ratio
EFW: Estimated fetal weight
HFSUH: Hiwot Fana Specialized University Hospital
IHRERC: Institutional Health Research Ethics Review Committee
NICU: Neonatal Intensive Care Unit
SVD: Spontaneous Vaginal Delivery
VIF: variance inflation factor
WHO: World health organization.
